# Case-Control Study and Meta-Analysis Show a Weak Association between ANTXR2 Polymorphisms and Ankylosing Spondylitis in Chinese Han

**DOI:** 10.1155/2018/8365173

**Published:** 2018-09-05

**Authors:** Jiayue Hu, Liping Du, Wencheng Su, Shengyun Liu, Jing Deng, Qinfeng Cao, Gangxiang Yuan, Aize Kijlstra, Peizeng Yang

**Affiliations:** ^1^The First Affiliated Hospital of Chongqing Medical University, Chongqing Key Laboratory of Ophthalmology and Chongqing Eye Institute, Chongqing, China; ^2^Rheumatology Department, The First Affiliated Hospital of Zhengzhou University, Zhengzhou, China; ^3^University Eye Clinic Maastricht, Maastricht, Netherlands

## Abstract

Previous studies have demonstrated associations of ANTXR2 gene polymorphisms with ankylosing spondylitis (AS). These associations differ depending on the ethnic populations and AS subgroups studied. Purposes of the current study were to evaluate the associations of 4 single nucleotide polymorphisms (SNPs) of the ANTXR2 gene with susceptibility to AS alone or AS in combination with acute anterior uveitis (AAU) in Chinese Han. Therefore, a case-control association study was performed in 880 AS^+^AAU^−^, 860 AS^+^AAU^+^, and 1700 healthy controls. Genotyping was performed using the iPLEXGold genotyping assay. Our results showed a weak association of rs6534639 AA genotype with AS^+^AAU^+^ patients (p=0.042), which was lost after correction for multiple comparisons. No other association was found between SNPs of ANTXR2 and susceptibility of AS^+^AAU^−^ or AS^+^AAU^+^. A meta-analysis was performed to evaluate the associations of polymorphisms in the ANTXR2 gene with AS. Results showed a weak association of rs4389526 with AS susceptibility in all studies but failed to show an association of rs6534639 with AS in Chinese Han. Taken together, this study shows no association between ANTXR2 polymorphisms and AS susceptibility in a Chinese Han population, but meta-analysis showed that rs4389526 in the ANTXR2 gene was weakly associated with AS susceptibility in both Caucasian and Chinese Han patients.

## 1. Introduction

Ankylosing spondylitis (AS) is a chronic, inflammatory arthritis that primarily affects the spine and sacroiliac joints. It is now classified into the family of spondyloarthropathies. The disease may result in fusion and rigidity of the spine if no effective treatment is administrated. Acute anterior uveitis (AAU) is one of the most common complications of AS and it occurs in 30-40% of individuals with AS. Though the precise pathogenesis of AS and the involvement of the eye in AS is not clear, twins [1] and family [2] studies have shown that the genetic background is one of the most important risk factors. In addition to HLA-B27, which is the strongest genetic factor associated with AS and AAU, genome-wide association studies (GWAS) have identified various other loci contributing to susceptibility to AS, such as interleukin-23 receptor, endoplasmic reticulum associated aminopeptidase (ERAP1/ERAP2), runt-related transcription factor 3 (RUNX3), and anthrax toxin receptor 2 (ANTXR2) [3–5]. Furthermore, certain susceptibility genes show an association only in AS patients without AAU (AS^+^AAU^−^) and not in AS patients with AAU (AS^+^AAU^+^), suggesting that these genes may be specific for isolated AS [6, 7].

ANTXR2 encodes a receptor for anthrax toxin and may be involved in extracellular matrix adhesion. Mutations in this gene cause juvenile hyaline fibromatosis and infantile systemic hyalinosis [8, 9]. Though the precise mechanism of ANTXR2 involvement in AS is not clear, several studies showed an association of genetic polymorphisms in this gene with AS [3, 6, 10]. Discrepancies have been noted, whereby two studies failed to find an association of this gene with AS in Chinese patients [11, 12], indicating that the association of ANTXR2 with AS may depend on the ethnic background. Furthermore, Robinson et al. [6] reported that rs4389526 in the ANTXR2 gene showed an association with AS^+^AAU^−^ but not with AS^+^AAU^+^ suggesting a different genetic predisposition for the two AS subgroups. Since the ANTXR2 associations have not yet been investigated in Chinese AS subgroups we decided to perform a case-control study in a large group of AS^+^AAU^−^ and AS^+^AAU^+^ patients.

## 2. Methods

### 2.1. Case and Control Cohorts

A total of 880 unrelated Chinese Han patients with AS without AAU (AS^+^AAU^−^) and 860 AS with AAU (AS^+^AAU^+^) were recruited from the Rheumatology Department of the First Affiliated Hospital of Zhengzhou University (Zhengzhou, China) and the Ophthalmic Center of the First Affiliated Hospital of Chongqing Medical University (Chongqing, China) from February 2010 to August 2016. All AS patients were diagnosed according to the 1984 modified New York criteria [13]. At the same time, a total of 1700 geographically and ethnically matched Chinese Han healthy individuals (≥18 years old; no systemic diseases, autoimmune diseases, and chronic back pains) were also enrolled. Furthermore, the controls were matched by age and sex. The study complied with the Declaration of Helsinki and was supported by the local ethical research committee of the Chongqing Medical University. Informed consent was taken from all participants.

### 2.2. Genomic DNA Preparation, SNP Selection, and Genotyping

**Figure 1:**
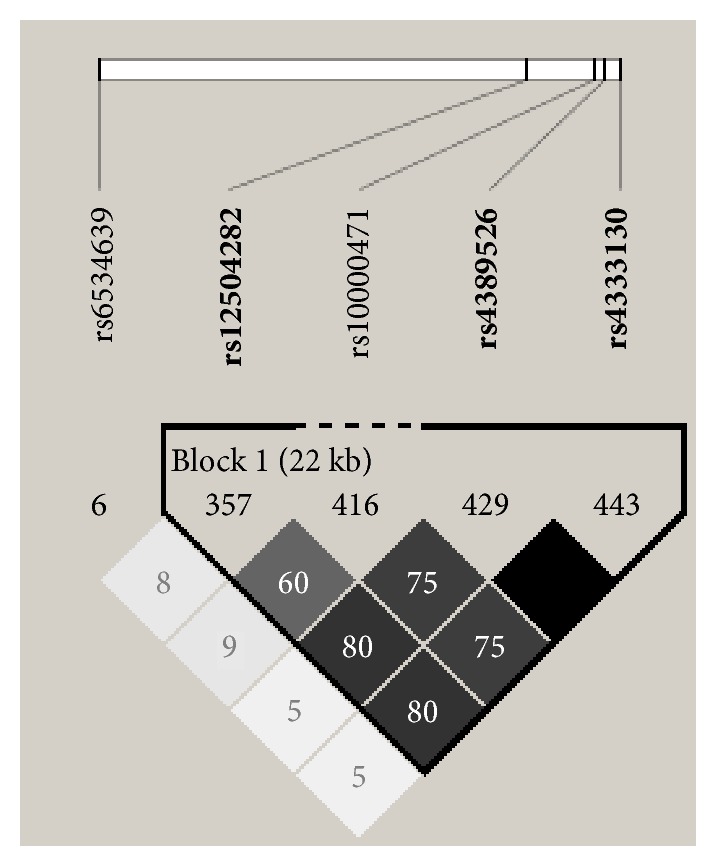
Strong linkage disequilibrium (LD) between SNP rs4333130 and rs4389526 (r2=1) based on HapMap 3 CHB (Chinese from Beijing) (HaploView4.2).

Genomic DNA was extracted and purified from peripheral blood using the QIAamp DNA Blood Mini Kit (250) (QIAGEN, Valencia, CA, USA). DNA concentration was measured with Nanodrop 2000 equipment (Thermo Fisher Scientific, Wilmington, DE, USA). Standardized and quality checked DNA was stored at-40°C until used. Five most common candidate SNPs (rs10000471, rs6534639, rs4389526, rs12504282, and rs4333130) were selected according to studies reported previously [14]. The SNPs rs4333130 and rs4389526 were demonstrated to be in strong linkage disequilibrium (LD) with each other (r^2^=1) according to the HAPMAP dataset and only rs4389526 was therefore included in the present study (Figure 1). The iPLEX Gold Genotyping Assay technique was used for genotyping analysis on a Sequenom MassArray System (Sequenom, San Diego, Calif). MassArray Assay Design software (Sequenom, San Diego, CA) was applied for primer design [19]. The study was performed strictly following the standard procedures of the manufacturer (Agena Bioscience, California, USA).

### 2.3. Statistical Analysis

Allele and genotype frequencies were calculated with the Sequenom MassArray System platform. The *X*^2^ test was applied for Hardy-Weinberg equilibrium (HWE) analysis for all tested SNPs. P values were corrected for multiple comparisons (Bonferroni adjustment according to the number of comparisons). A corrected P value (Pc) of less than 0.05 was considered to be statistically significant. SPSS (SPSS Inc., Chicago, Illinois, USA) version 19.0 was used to calculate 95% confidence intervals (CI) and odds ratios (ORs).

### 2.4. Meta-Analysis

A literature search was performed using PubMed for studies on the associations of polymorphisms in the ANTXR2 gene with AS. “AS” or “ankylosing spondylitis”, “acute anterior uveitis” or “AAU”, “ANTXR2”, and “polymorphism” were used as combined keywords in the search in the PubMed database (up to October 2017). The following inclusion criteria were used: (1) case-control studies on AS susceptibility evaluating ANTXR2 polymorphisms, (2) contained data of MAF (Minor Allele Frequencies), 95%CI and OR, and (3) showed numbers of cases and controls. References in the studies included were investigated to exclude duplicate data. Replication studies and twins or family studies were excluded. The following data were collected from each study: author, year of publication, ethnicity, numbers of controls and cases, and MAF of polymorphisms in theANTXR2 gene. RevMan5.0 (http://ims.cochrane.org/revman/download) software was applied for the meta-analysis. Both the fixed and random effect models were used (*I*^2^ statistics). The* I*^2^ index was used to quantify the degree of heterogeneity among different studies.

## 3. Results

### 3.1. Case-Control Study

Demographic features of AS^+^AAU^−^ and AS^+^AAU^+^ patients are shown in Table 1. There was no difference in the male frequency between the AS^+^AAU^−^ and AS^+^AAU^+^ groups (67.56% versus 69.32%, p=0.43). The HLA-B27^+^ (96.93%) status in AS^+^AAU^−^was significantly higher than that (84.30%) observed in the AS^+^AAU^+^ group (p < 0.001). The average age of AS^+^AAU^+^ patients was significantly higher than that of AS^+^AAU^−^ patients. Four SNPs were genotyped in1740 AS patients with or without AAU (AS^+^AAU^−^ 880, AS^+^AAU^+^ 860) and 1700 healthy controls. All of the tested polymorphisms in the ANTXR2 gene are shown in Table 2. Genotype frequencies of all SNPs were in line with the HWE (both Fisher's p value and Pearson's p value>0.05).

When comparing total AS patients (including AS^+^AAU^−^ and AS^+^AAU^+^ patients) with controls, there was a trend towards a lower rs4389526 T allele frequency in AS patients (p=0.054). There were no differences in the allele and genotype frequencies of the other three SNPs tested in the present study between total AS patients and controls. When comparing AS^+^AAU^−^ patients with controls, there were also no differences in the allele and genotype frequencies of the four SNPs tested between patients and controls, except for a trend towards a lower rs4389526 T allele frequency in AS^+^AAU^−^ patients (p=0.052). When comparing AS^+^AAU^+^ patients with controls, no significant differences were observed for rs12504282, rs10000471, and rs4389526 between the two groups. The frequency of the rs6534639 AA genotype of in AS^+^AAU^+^ patients was slightly higher than that seen in controls (p=0.042). However, this significance was lost after correction for multiple comparisons (Table 2). Haplotype analysis and stratified analysis (stratified by HLA-B27 and sex) failed to find any haplotype or SNP associated with AS and its subgroups (data not shown).

### 3.2. Meta-Analysis

Literature search in PubMed identified 12 studies on the association of ANTXR2 with AS. Three studies were excluded because they did not include all data we defined in the inclusion criteria. Finally, 9 studies met the study inclusion criteria, including 4 studies from Caucasian populations and 5 studies from Asian populations (1 from Korea, 4 from China including the present study) (Table 3). There were 3 independent groups of data in the Evans study [3] and two groups in the Reveille's study [18]. Data from the PubMed SNP database showed that the frequency of ANTXR2/rs4333130 C allele is 39-45% in Europe but only 0.05-0.1% in Asia (including Chinese, Korean and Japanese). These results were consistent with the complete LD of rs4333130 with rs4389526 based on HapMap database 3 (CHB).

**Figure 2:**
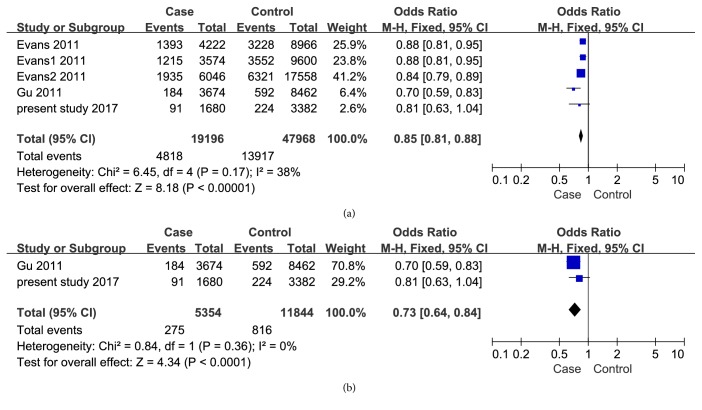
Forest plots for ANTXR2/rs4389526 and AS susceptibility. (a) All studies from Caucasian and China, I^2^= 38% and p<0.00001. (b) Studies from China, I^2^= 0% and p<0.0001. Events represent the total number of rs4389526 T allele. Total represents the total number of rs4389526 A and T alleles.

**Figure 3:**
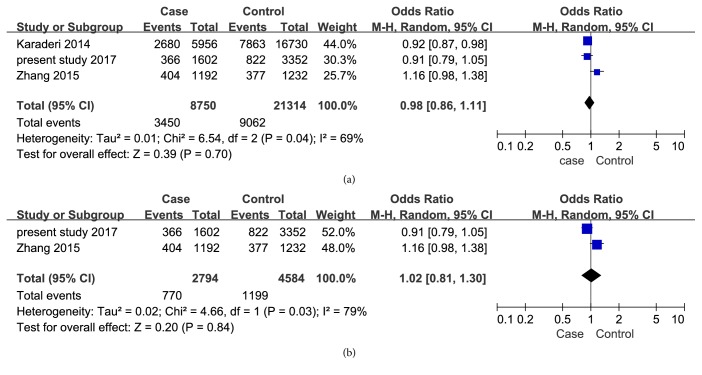
Forest plots for ANTXR2/rs6534639 and AS susceptibility. (a) Studies from both Caucasian and China, I^2^= 69%, p=0.70. (b) Studies from Chinese population, I^2^= 79%, p=0.84. Events represent the total number of rs6534639 A allele. Total represents the total number of rs6534639 A and C alleles.

The meta-analysis showed an association of rs4389526 with AS susceptibility in all studies as well as in the studies with Chinese Han patients (p<0.00001, OR: 0.85 (0.81-0.88),* I*^2^= 38%; p<0.0001, OR: 0.73 (0.64-0.84),* I*^2^= 0%) (Figure 2). Our meta-analysis did not show an association of rs6534639 with AS in Chinese Han (p=0.84, OR: 1.02 (0.81-1.30),* I*^2^=79%) (Figure 3). Heterogeneity (*I*^2^) was found in studies concerning the other 3 SNPs investigated. Obvious heterogeneity was found in studies on the association of rs4333130 with AS susceptibility in Caucasian populations. Random combinatorial models were used when analyzing the various studies from Caucasian and Asia. The study from Karaderi [14] and Chen [11] were major contributors to heterogeneity. The funnel plot of the loci showed no significant asymmetry. This shows that our meta-analysis did not have a significant publication bias.

## 4. Discussion

This study did not show an association of ANTXR2 gene polymorphisms with AS alone or in combination with AAU in Chinese Han patients. These results confirm earlier data in Chinese Han patients with AS [11, 12] and are new with respect to the fact that we made a separate analysis for patients with or without AAU. Our results showed that the AA genotype frequency of rs6534639 was slightly higher than that in controls and that there was also a trend towards a lower rs4389526 T allele frequency in AS patients. However, significance was lost after correction for multiple comparisons. A meta-analysis using 9 studies showed an association of rs4389526 with AS susceptibility both in all studies and when combining the studies from China, including our data. No significant association was found for the other SNPs investigated. This shows that the association is weak and that statistical significance is only reached by obtaining a very large sample size.

In the present study, the rs4389526T allele showed a trend towards a lower frequency in AS^+^AAU^−^ patients but not in AS^+^AAU^+^ patients, suggesting that this SNP might be a specific AS protective locus. These data are in agreement with an earlier study with Caucasian patients, demonstrating that rs4389526 in the ANTXR2 gene had an effect only in AS patients without AAU but not in AS patients with AAU [6]. SNP rs4389526 is in complete LD with rs4333130, which was mainly examined in other studies [11, 12, 14, 20]. Our meta-analysis (data not shown) also showed an association of rs4333130 with AS susceptibility both in Caucasian and Asian populations (p<0.00001, OR: 0.81 (0.81-0.87),* I*^2^=17%; p=0.008, OR: 0.76 (0.63-0.93),* I*^2^=0%). These two SNPs are very close to each other and are located near a putative transcription factor-binding region, suggesting that this region may contribute to AS susceptibility by affecting ANTXR2 expression [14].

Our results also demonstrated a weak association for the rs6534639 genotype AA with the AS^+^AAU^+^ group but not with the AS^+^AAU^−^ group. The rs6534639 genotype CC was reported to be associated with AS by a previous study from China [15]. Due to the obvious heterogeneity, the meta-analysis failed to find an association of this SNP with AS in all studies or in the Chinese population. SNP rs12504282 was reported to be strongly associated with AS by one Caucasian study [14]. However, there was no association of this SNP with AS with or without AAU in the present study. Two other studies from China did not examine this SNP [11, 12]. SNP rs10000471 has also been shown to be associated with AS in one previous study [14] but this could not be confirmed in Chinese Han.

Our study has a number of limitations. The patients included in the study came from two hospitals, and a selection bias for certain types of AS^+^AAU^+^ and AS^+^AAU^−^ may exist considering the vast amount of Han Chinese in Mainland China. A limitation of our meta-analysis is that two SNPs, namely, rs12504282 and rs10000471, were only evaluated in two studies [14]. Further multicenter and multiethnic studies testing more SNPs are therefore needed to describe the exact role of ANTXR2 in the susceptibility to AS alone or in combination with AAU. For stratified analysis, it should be noted that 1700 controls were used as the control group for each subtype because the HLA-B27 distribution is not clear for controls. Another limitation is that we cannot exclude that some of our patients from the AS^+^AAU^−^ group may develop AAU later in life. Given the age of our patients and the dynamics of the disease we do not expect that this will be a large number.

In conclusion, ANTXR2 polymorphisms showed a weak association with AS susceptibility in Chinese Han population. Meta-analysis showed that ANTXR2 polymorphisms in the rs4389526 locus are associated with AS susceptibility in both the Caucasian as well as in the Chinese Han population.

## Figures and Tables

**Table 1:** HLA-B27, sex, history of AS, number, and age of AS and AS^+^AAU^+^ patients.

**Phenotype**	**Total**	%
AS^+^AAU^−^ patients	880	
Mean age (year ±S.D.)a		23.91±2.77
History of AS (year±S.D.)b		4.91±3.32
Mean present age (year ±S.D.)		28.82±2.92
Male	610	69.32
Female	270	30.68
HLA-B27^+^	853	96.93
AS^+^AAU^+^ patients	860	
Mean age (year ±S.D.)a		33.1±7.64
Mean age (year ±S.D.)c		30.0±9.67
History of AS (year ±S.D.)b		5.51±3.76
Male	581	67.56
Female	279	32.44
HLA-B27^+^	725	84.3
Controls	1700	
Mean age (year ±S.D.)		25.51±6.24
Male	1171	68.88
Female	529	31.12

a: the average age at the time of AS diagnosis.

b: the average number of years of having AS.

c: the average age at the time of AAU diagnosis.

**Table 2:** Allele and genotype frequencies of ANTXR2 SNPs in Chinese Han AS^+^AAU^−^ patients, AS^+^AAU^+^ patients, and controls.

**SNPs**	**Genotype**	**Control**	**AS** ^**+ **^ **AAU** ^−^	**P value**	**Pc**	**OR(95**%**CI)**	**AS** ^**+ **^ **AAU** ^**+**^	**P value**	**Pc**	**OR(95**%**CI)**
rs6534639	CC	955	481	0.08	NS	0.88(0.72-1.05)	455	0.41	NS	1.03(0.86-1.22)
	CA	620	274	0.096	NS	1.13(0.95-1.35)	294	0.297	NS	1.06(0.88-1.27)
	AA	101	46	0.429	NS	1.05(0.74-1.51)	53	0.042	NS	0.70(0.48-1.03)
	A allele	822	366	0.104	NS	0.91(0.79-1.05)	400	0.191	NS	1.07(0.93-1.24)

rs4389526	AA	1469	751	0.055	NS	0.80(0.62-1.04)	698	0.1	NS	0.83(0.64-1.09)
	AT	210	87	0.069	NS	1.23(0.95-1.61)	84	0.119	NS	1.19(0.91-1.55)
	TT	7	2	0.379	NS	1.75(0.36-8.43)	2	0.417	NS	1.63(0.34-7.87)
	T allele	224	91	0.052	NS	0.81(0.61-1.04)	88	0.096	NS	0.84(0.65-1.08)

rs12504282	TT	1406	712	0.195	NS	1.11(0.89-1.37)	714	0.201	NS	0.90(0.72-1.13)
	TC	274	154	0.187	NS	0.90(0.71-1.12)	129	0.282	NS	1.09(0.86-1.35)
	CC	12	6	0.585	NS	1.03(0.39-2.76)	5	0.476	NS	1.20(0.42-3.43)
	C allele	298	166	0.214	NS	1.09(0.89-1.33)	139	0.25	NS	0.92(0.75-1.14)

rs10000471	CC	6	2	0.46	NS	1.53(0.31-7.57)	1	0.261	NS	3.03(0.36-25.17)
	CT	172	93	0.326	NS	0.93(0.71-1.22)	81	0.33	NS	1.08(0.82-1.42)
	TT	1496	755	0.437	NS	1.03(0.79-1.34)	760	0.267	NS	0.91(0.67-1.20)
	C allele	184	97	0.402	NS	0.96(0.75-1.24)	83	0.218	NS	1.12(0.86-1.46)

**Table 3:** Five candidate SNPs in ANTXR2 reported by previous studies.

**SNP**	**Dis**	**Ethnic groups**	**Reference**
rs6534639	AS	Chinese/Caucasian	Karaderi et al. [14]; Zhang et al. [15]
rs4333130	AS	Chinese/Korean/Caucasian	Momenzadeh et al. [16]; Zhang et al. [15]; Karaderi et al. [14]; Bang et al. [17]; Chen et al. [11]; Guo et al. [12]; Reveille et al. [18]
rs4389526	AS	Chinese/Caucasian	Evans et al. [3]; Lin et al. [10];
rs12504282	AS	Caucasian	Karaderi et al. [14]
rs10000471	AS	Caucasian	Karaderi et al. [14]

## Data Availability

The data used to support the findings of this study are available from the corresponding author upon request.

## Additional Points

*Key Messages. *A case-control study shows that ANTXR2 polymorphisms are not significantly associated with AS susceptibility in Chinese Han. Meta-analysis showed that ANTXR2 polymorphisms were weakly associated with AS susceptibility in both Caucasian and Chinese Han.

## References

[1] Braun J., Bollow M., Remlinger G. (1998). Prevalence of spondylarthropathies in HLA-B27 positive and negative blood donors. *Arthritis & Rheumatology*.

[2] Tsui F. W. L., Haroon N., Reveille J. D. (2010). Association of an ERAP1 ERAP2 haplotype with familial ankylosing spondylitis. *Annals of the Rheumatic Diseases*.

[3] Evans D. M., Spencer C. C., Pointon J. J. (2011). Interaction between ERAP1 and HLA-B27 in ankylosing spondylitis implicates peptide handling in the mechanism for HLA-B27 in disease susceptibility. *Nature Genetics*.

[4] Australo-Anglo-American Spondyloarthritis C., Reveille J. D., Sims A. M. (2010). Genome-wide association study of ankylosing spondylitis identifies non-MHC susceptibility loci. *Nature Genetics*.

[5] International Genetics of Ankylosing Spondylitis C., Cortes A., Hadler J. (2013). Identification of multiple risk variants for ankylosing spondylitis through high-density genotyping of immune-related loci. *Nature Genetics*.

[6] Robinson P. C., Claushuis T. A., Cortes A. (2015). Genetic dissection of acute anterior uveitis reveals similarities and differences in associations observed with ankylosing spondylitis. *Arthritis Rheumatol*.

[7] Robinson P. C., Leo P. J., Pointon J. J. (2016). The genetic associations of acute anterior uveitis and their overlap with the genetics of ankylosing spondylitis. *Genes & Immunity*.

[8] Hanks S., Adams S., Douglas J. (2003). Mutations in the gene encoding capillary morphogenesis protein 2 cause juvenile hyaline fibromatosis and infantile systemic hyalinosis. *American Journal of Human Genetics*.

[9] Denadai R., Raposo-Amaral C. E., Bertola D. (2012). Identification of 2 novel ANTXR2 mutations in patients with hyaline fibromatosis syndrome and proposal of a modified grading system. *American Journal of Medical Genetics Part A*.

[10] Lin Z., Bei J. X., Shen M. (2011). A genome-wide association study in Han Chinese identifies new susceptibility loci for ankylosing spondylitis. *Nature Genetics*.

[11] Chen C., Zhang X., Wang Y. (2012). ANTXR2 and IL-1R2 polymorphisms are not associated with ankylosing spondylitis in Chinese Han population. *Rheumatology International*.

[12] Guo C., Xia Y., Yang Q., Qiu R., Zhao H., Liu Q. (2012). Association of the ANTXR2 gene polymorphism and ankylosing spondylitis in Chinese Han. *Scandinavian Journal of Rheumatology*.

[13] van der Linden S., Valkenburg H. A., Cats A. (1984). Evaluation of diagnostic criteria for ankylosing spondylitis. A proposal for modification of the New York criteria. *Arthritis & Rheumatology*.

[14] Karaderi T., Keidel S. M., Pointon J. J. (2014). Ankylosing spondylitis is associated with the anthrax toxin receptor 2 gene (ANTXR2). *Annals of the Rheumatic Diseases*.

[15] Zhang Z., Yu K., Dai D. (2016). A complex role of anthrax toxin receptor 2 polymorphisms and capillary morphogenesis protein 2 in ankylosing spondylitis pathogenesis. *Clinical Rheumatology*.

[16] Momenzadeh P., Mahmoudi M., Beigy M. (2016). Determination of IL1 R2, ANTXR2, CARD9, and SNAPC4 single nucleotide polymorphisms in Iranian patients with ankylosing spondylitis. *Rheumatology International*.

[17] Bang S. Y., Kim T. H., Lee B. (2011). Genetic studies of ankylosing spondylitis in Koreans confirm associations with ERAP1 and 2p15 reported in white patients. *Journal of Rheumatology*.

[18] Reeves C. V., Dufraine J., Young J. A. T., Kitajewski J. (2010). Anthrax toxin receptor 2 is expressed in murine and tumor vasculature and functions in endothelial proliferation and morphogenesis. *Oncogene*.

[19] Su W., Du L., Liu S. (2018). ERAP1/ERAP2 and RUNX3 polymorphisms are not associated with ankylosing spondylitis susceptibility in Chinese Han. *Clinical & Experimental Immunology*.

[20] Ou Y. (2015). Anthrax toxin receptor 2 gene (ANTXR2) rs4333130 is associated with ankylosing spondylitis. *International Journal of Clinical and Experimental Medicine*.

